# AAV1.NT-3 gene therapy prevents age-related sarcopenia

**DOI:** 10.18632/aging.204577

**Published:** 2023-03-09

**Authors:** Burcak Ozes, Lingying Tong, Morgan Myers, Kyle Moss, Alicia Ridgley, Zarife Sahenk

**Affiliations:** 1 Center for Gene Therapy, The Abigail Wexner Research Institute, Nationwide Children’s Hospital, Columbus, OH 43205, USA; 2 Department of Pediatrics and Neurology, Nationwide Children’s Hospital and The Ohio State University, Columbus, OH 43205, USA; 3 Department of Pathology and Laboratory Medicine, Nationwide Children’s Hospital, Columbus, OH 43205, USA

**Keywords:** sarcopenia, gene therapy, aging, NT-3, muscle remodeling

## Abstract

Sarcopenia is progressive loss of muscle mass and strength, occurring during normal aging with significant consequences on the quality of life for elderly. Neurotrophin 3 (NT-3) is an important autocrine factor supporting Schwann cell survival and differentiation and stimulating axon regeneration and myelination. NT-3 is involved in the maintenance of neuromuscular junction (NMJ) integrity, restoration of impaired radial growth of muscle fibers through activation of the Akt/mTOR pathway. We tested the efficacy of NT-3 gene transfer therapy in wild type (WT)-aged C57BL/6 mice, a model for natural aging and sarcopenia, via intramuscular injection 1 × 1011 vg AAV1.tMCK.NT-3, at 18 months of age. The treatment efficacy was assessed at 6 months post-injection using run to exhaustion and rotarod tests, *in vivo* muscle contractility assay, and histopathological studies of the peripheral nervous system, including NMJ connectivity and muscle. AAV1.NT-3 gene therapy in WT-aged C57BL/6 mice resulted in functional and *in vivo* muscle physiology improvements, supported by quantitative histology from muscle, peripheral nerves and NMJ. Hindlimb and forelimb muscles in the untreated cohort showed the presence of a muscle- and sex-dependent remodeling and fiber size decrease with aging, which was normalized toward values obtained from 10 months old WT mice with treatment. The molecular studies assessing the NT-3 effect on the oxidative state of distal hindlimb muscles, accompanied by western blot analyses for mTORC1 activation were in accordance with the histological findings. Considering the cost and quality of life to the individual, we believe our study has important implications for management of age-related sarcopenia.

## INTRODUCTION

Sarcopenia is a common geriatric syndrome, defined as generalized, progressive loss of muscle mass and strength, occurring during normal aging with significant consequences on the quality of life for the elderly [1–4]. Age-related degradation of muscle leads to a slow but continuous loss in lean mass, starting after the age of 40 and thereafter is accelerated by age 70 [5, 6]. The prevalence of sarcopenia between age 60 to 70 is reported to be 5–13% but increases to 11–50% in people older than 80 [7, 8]. Sarcopenic patients face decreased strength and mobility, therefore are at higher risk for falls, bone fractures and increased mortality. Since the number and proportion of the global aging population is rapidly growing, preventing or delaying sarcopenia should have a great impact on the quality of life for elderly, as well as its socio-economic burden on individuals and society. Current strategies are mainly focused on exercise and nutrition, and despite some molecular pathways involved in aging have been suggested with potential for drug development, no specific medications have been developed with therapeutic impact in sarcopenia.

Underlying pathophysiologic mechanisms contributing to sarcopenia are highly complex and multifactorial. In addition to behavioral or “extrinsic” factors, such as a sedentary lifestyle, the remaining contributing/causal factors appear directly related to the aging process of muscle to include mitochondrial dysfunction with oxidative damage, lysosomal dysfunction, decreased protein synthesis, decreased anabolic hormones, inflammaging/immune-senescence and satellite cells dysfunction (decreased number and regenerative capacity). Mitochondria, as the “power-house” of cells, is considered as a central player in overall aging process, and certainly in muscle going through sarcopenic change. In a recent study using neurotrophin 3 (NT-3) gene therapy, we demonstrated that NT-3 increases muscle fiber diameter through direct activation of mTORC1 pathway [9]. In addition, NT-3 was shown to improve hypomyelination state, neuromuscular junction (NMJ) connectivity, have anti-inflammatory, antioxidant, antiapoptotic properties, and enhances mitochondria biogenesis [10–14]. This suggests a potential application of NT-3 gene therapy for muscle wasting conditions including age-related sarcopenia. In this study, we used a triple muscle-specific creatine kinase (tMCK) promoter to restrict NT-3 expression to the skeletal muscle and self-complimentary adeno-associated virus serotype 1 (scAAV1) as vector to assess the therapeutic efficacy of AAV1.NT-3 in wild type-aged C57BL/6J mice, a model for natural aging and sarcopenia. Quantitative histopathologic parameters served to address age-related changes in muscle, peripheral nerve and NMJ. These changes include muscle fiber size and fiber type switch, myelin thickness and the innervated status of the NMJ. Functional studies and *in vivo* muscle physiology were used to assess the motor strength of the mice. The results show that AAV1.NT-3 gene therapy in the wild type C57BL/6J mice at 2 years of age, unequivocally improved the function of sarcopenic muscle, increased muscle fiber size, myelin thickness and NMJ connectivity. In addition, we found attenuation of age-related kyphosis and coat changes as well.

## RESULTS

### rAAV.NT-3 vector production and potency

scAAV1.tMCK.NT-3 design (Supplementary Figure 1A), and production followed previously described methods at Nationwide Children`s Hospital, Columbus [10]. scAAV1.tMCK.NT-3, at 1 × 10^11^ vg dose, was delivered to the gastrocnemius muscle of 18 months old C57BL/6 mice. Blood samples from terminally anesthetized treated and untreated mice were collected by cardiac puncture at six months post gene injection, and serum was assayed for NT-3 levels using a capture ELISA, as previously reported [10]. No detectable NT-3 levels were found in the untreated C57BL/6 mice at 2 years of age, in contrast to the treated cohort (Supplementary Figure 1B), showing significant improvements in functional and histologic outcome measures as described below. Similar to our previous observations in younger age groups, we found no effect of sex on serum NT-3 levels in the aged C57BL/6 mice.

### NT-3 gene therapy improved function, attenuated age-related musculoskeletal and skin changes in the aged C57BL/6 mouse

**Figure 1:**
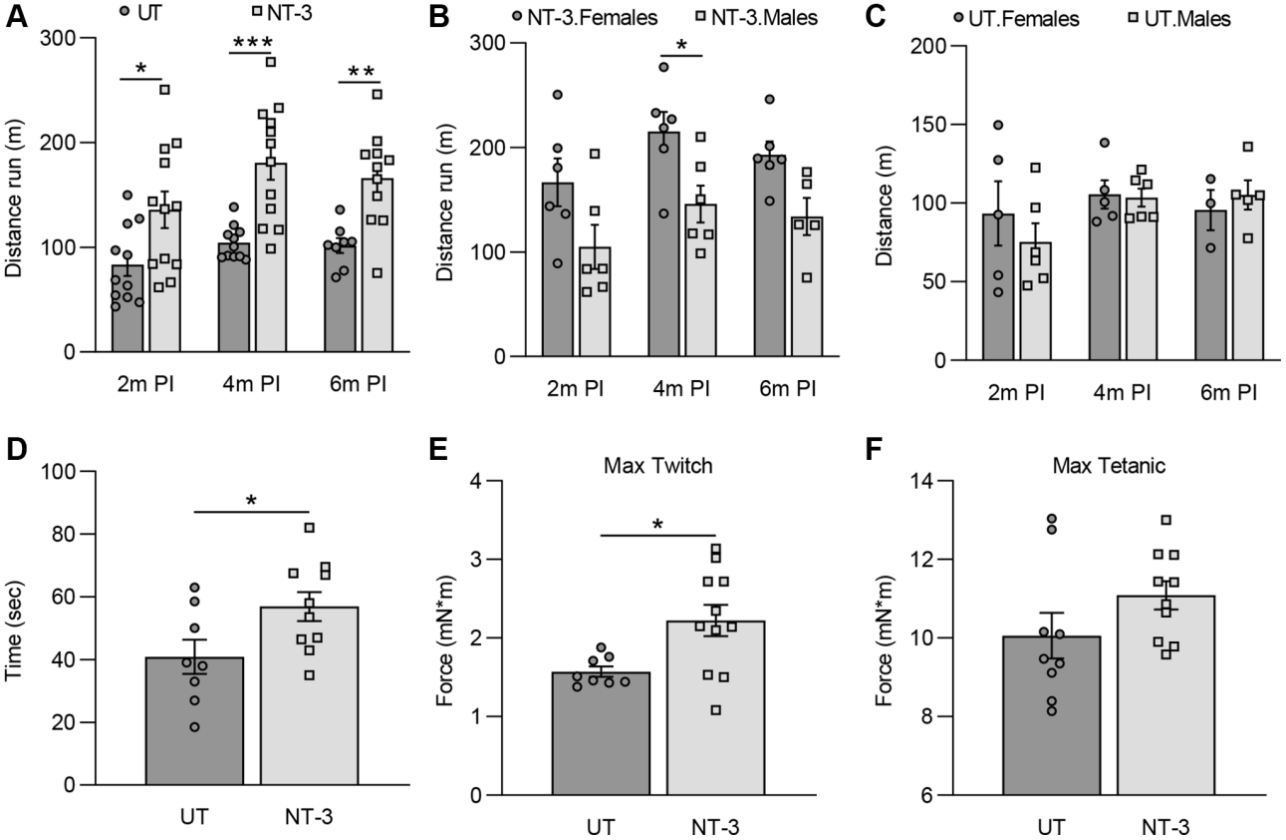
**Functional and *in vivo* muscle physiology improvements in 2-year-old C57BL/6 mice with AAV1.NT-3 gene transfer therapy.** (**A**–**C**) Treadmill performance test performed at 2-, 4-, and 6-months post-injection (PI). (**A**) AAV1.NT-3 treated mice showed significant improvement at the time points tested (2 months PI, NT-3: 135.8 m, *n* = 12 vs. UT: 83.5 m, *n* = 11, *p* = 0.0184; 4 months PI, NT-3: 180.6 m, *n* = 12 vs. UT: 104.4 m, *n* = 11, *p* = 0.0003; 6 months PI, NT-3: 166.1 m, *n* = 11, vs. UT: 101.6 m, *n* = 8; *p* = 0.0077). (**B**) Treadmill performance of the female mice was better than the males in the treated cohort (2 months PI, F: 166.7 m, *n* = 6 vs. M: 104.9 m, *n* = 6; 4 months PI, F: 215.3 m, *n* = 6 vs. M: 145.9 m, *n* = 6, *p* = 0.0394; 6 months PI, F: 192.9 m, *n* = 6, vs. M: 133.9 m, *n* = 5) while no sex effect was observed in the (**C**) untreated cohort (2 months PI, F: 93.4 m, *n* = 5 vs. M: 75.3 m, *n* = 6; 4 months PI, F: 105.5 m, *n* = 5 vs. M: 103.5 m, *n* = 6; 6 months PI, F: 95.6 m, *n = 3*, vs. M: 105.2 m, *n* = 5). (**D**) AAV1.NT-3 treated mice showed significant improvement in the rotarod at end point (NT-3: 56.9 sec, *n* = 10 vs. UT: 40.9 sec, *n* = 8, *p* = 0.0374). *In vivo* muscle contractility assay showed a higher force output in (**E**) maximum twitch response in the treated cohort whereas (**F**) increase in the maximum tetanic measurement did not reach significance levels (Max twitch, NT-3: 2.22 mN*m, *n* = 11 vs. UT: 1.57 mN*m, *n* = 8, *p* = 0.0149; Max tetanic, NT-3: 11.09 mN*m, *n* = 10 vs. UT: 10.06 mN*m, *n* = 9). Data is represented as mean ± SEM; Two-way ANOVA, Sidak’s multiple comparisons test for (**A**–**C**) and student *t*-test for (**D**–**F**); ^*^p < 0.05, ^**^p < 0.01, ^***^p < 0.001, ^****^p < 0.0001.

Run to exhaustion treadmill test was used to assess the efficacy of NT-3 gene therapy on motor function at 2-, 4- and 6-months post gene delivery. AAV1.NT-3 improved treadmill performance, leading to remarkable increases in running distances and time until exhaustion compared to the untreated counterparts at all time points tested. We found a 63% (NT-3, 135.8 ± 17.5, *n* = 12 vs. UT, 83.5 ± 11.1, *n* = 11; *p* = 0.0184), 73% (NT-3, 180.6 ± 16.2, *n* = 12 vs. UT, 104.4 ± 4.8, *n* = 11; *p* = 0.0003) and 63.5% (NT-3, 166.1 ± 13.8 *n* = 11, vs. UT, 101.6 ± 7.2, *n* = 8; *p* = 0.0077) increase in distance run at 2-, 4- and 6-months post gene delivery, respectively (Figure 1A). Treated females performed significantly better at 4 months post-injection (*p* = 0.0394) compared to treated males (Figure 1B), while there was no sex difference found in the untreated cohort (Figure 1C). We also assessed the efficacy of NT-3 gene therapy on motor coordination of aged C57BL/6 mice with rotarod test. NT-3 treated cohort displayed significantly better motor coordination with 39.2% longer duration compared to untreated at endpoint (NT-3, 56.9 ± 5.9, *n* = 10 vs. UT, 40.9 ± 6.9, *n* = 8; *p* = 0.038, Figure 1D). Treated females performed better than males without reaching statistical significance (Supplementary Figure 2A).

*In vivo* muscle contractility assay performed in the gastrocnemius muscle of C57BL/6 mice at endpoint showed an increase in the maximum twitch response with NT-3 gene therapy (NT-3, 2.22 ± 0.2, *n* = 11 vs. UT, 1.57 ± 0.1, *n* = 8; *p* = 0.015, Figure 1E) while the maximum tetanic response did not improve significantly (NT-3, 11.1 ± 0.4, *n* = 10 vs. UT, 10.1 ± 0.6, *n* = 9; *p* = 0.142, Figure 1F). When treatment effect on genders was analyzed, both the maximum twitch and maximum tetanic responses were found to be higher in males, without reaching statistical significance (Supplementary Figure 2B, 2C). The treatment did not alter the endpoint weight of the treated cohort (NT-3, *n* = 11, 37.0 ± 1.95 g; UT, *n* = 10, 37.6 ± 1.94 g; *p* = 0.84, Supplementary Figure 2D). Two untreated mice, aged 84 and 100 weeks, and one treated mouse, aged 99 weeks, died before reaching to the end point of the experiment.

We also documented improvements in age related musculoskeletal and skin changes in the NT-3 treated C57BL/6 mice including reduction of kyphosis, dermatitis, and alopecia (Supplementary Figure 3A). AAV1.NT-3 injected, and untreated cohorts were photographed at the end point, and the severity of kyphosis, dermatitis, and alopecia were assessed using a semiquantitative scoring system (0: none, 0.5: mild-moderate, 1: severe). Varying degrees of kyphosis were present in 83% of untreated mice, half of which were severe, while only 50% of the AAV1.NT-3 injected mice had kyphosis and one third were severe. Dermatitis, observed in 33% of untreated mice, was not seen in the treated group. Moderate to severe alopecia around the ears was present in 67% of the untreated mice, whereas this ratio decreased to 33% percent in treated mice (Supplementary Figure 3B–3D).

### Reversal of age-related neuromuscular histopathology with NT-3 gene therapy

**Figure 2:**
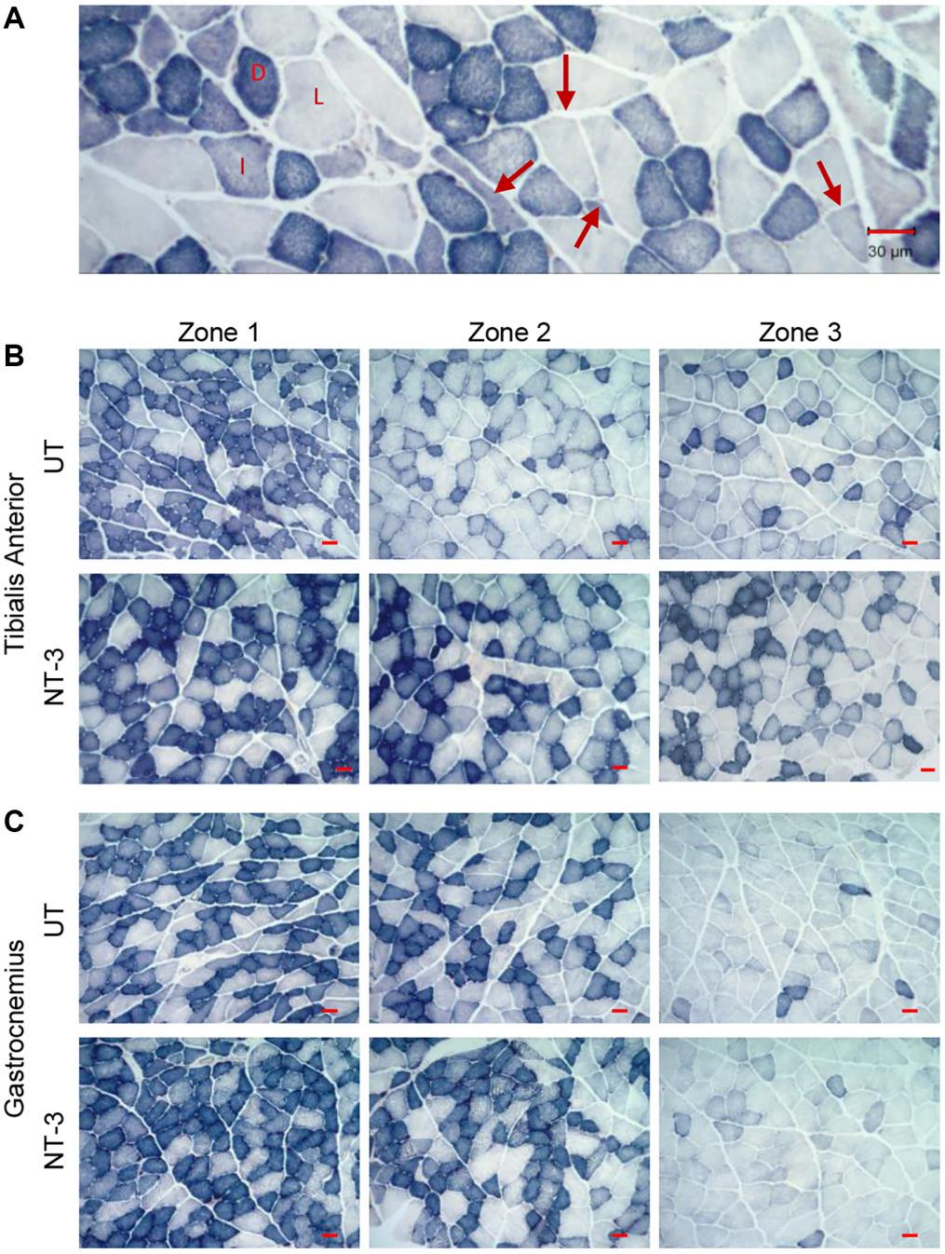
**Muscle fiber size increase in aged C57BL/6 mice with AAV1.NT-3 gene therapy.** (**A**) Succinic dehydrogenase (SDH)-stained skeletal muscle showing muscle fiber types based on mitochondria content and angular atrophic type 2 fibers (arrows). Dark (D) fibers are fatigue-resistant slow twitch oxidative (STO) or type1 fibers, intermediate (I)-stained fibers are fast twitch oxidative (FTO/type 2A) fibers, and light (L) fibers are fast twitch glycolytic (FTG/type 2B). Representative images from (**B**) tibialis anterior and (**C**) gastrocnemius muscles showing three different zones (deep, intermediate, and superficial; designated as Zone 1, 2, and 3) from the untreated (UT) and NT-3 treated cohorts. Scale bar = 30 μm.

We examined the efficacy of NT-3 gene therapy upon muscle fiber size and fiber type composition in tibialis anterior, gastrocnemius, quadriceps, and triceps muscles at 6 months post gene injection. Succinic dehydrogenase stain was used for quantification [15], which delineates fibers according to mitochondria content: fatigue-resistant slow-twitch oxidative (STO) fibers with darkest staining, the fast-twitch oxidative (FTO) with intermediate staining and the fast-twitch glycolytic (FTG) with lightest staining intensity (Figure 2A). Fiber size measurements were done on the representative images from deep, intermediate, and superficial zones of untreated and NT-3-treated samples as illustrated in the tibialis anterior and gastrocnemius muscles [15] (Figure 2B, 2C). Both muscles from the untreated cohort showed atrophic angular fibers with the majority belonging to fast twitch type 2 fibers, typical of sarcopenic muscle (Figure 2A). Compared to younger 10-month-old C57BL/6 mice, the mean muscle fiber size of all fiber types from the hindlimb muscles was smaller at 2 years of age, corroborating previous reports [16] and an overall fiber size increase was evident with treatment. Fiber type diameters (mean ± SEM μm; derived from the mean of measurements individually made in each mouse) from untreated and treated 2-year-old C57BL/6 mice of both sexes as combined, and separately for males and females, along with inclusion of data from the same strain at 10 months of age in all 4 muscles are detailed in Supplementary Figures 4–7 and Supplementary Tables 1–12.

**Figure 3:**
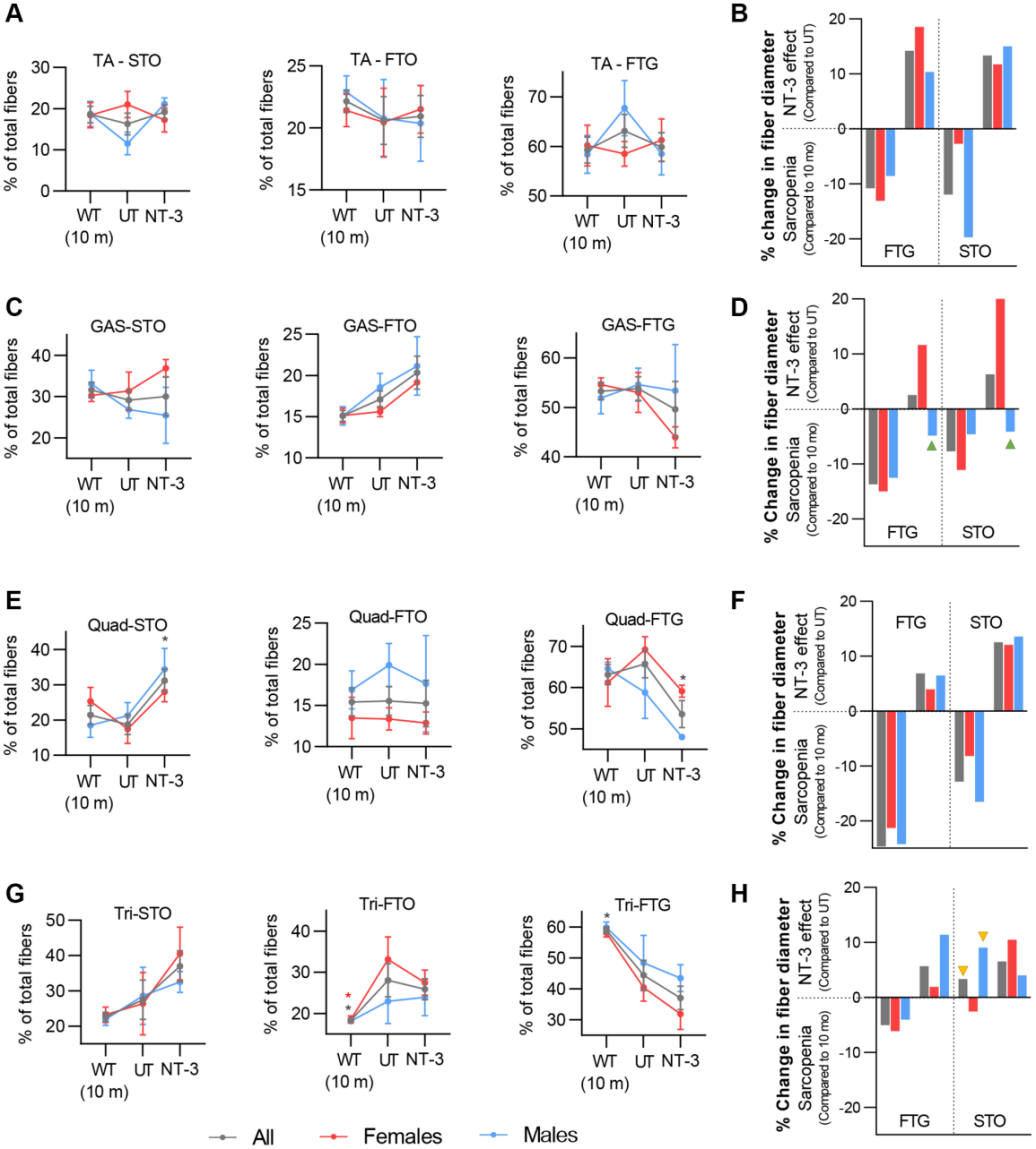
**NT-3 effect on fiber diameter and fiber type switch.** Line graphs represent the changes in fiber type contribution to total as percent (STO, FTO and FTG) in the muscles analyzed. Data are represented as mean ± SEM. Bar graphs represent percent changes in average fiber diameter of sarcopenic mice compared to 10-month-old mice to show age-related fiber size changes, and NT-3-treated mice compared to untreated 2-year-old mice to show the effect of treatment in average fiber size change. Mean fiber size is calculated for each cohort and percent change is determined based on these mean fiber size values for each muscle. Green arrow heads mark bars for treated mice showing no fiber size increase, yellow arrow heads depicting bars for untreated mice did show fiber size increase compared to 10-month- old mice, likely as compensatory change. (**A**, **B**) Tibialis anterior (UT, *n* = 8; NT-3, *n* = 9; WT, *n* = 8; with equal sex distribution), (**C**, **D**) gastrocnemius (UT, *n* = 6; NT-3, *n* = 6; WT, *n* = 8; with equal sex distribution) (**E**, **F**) quadriceps (UT, *n* = 6; NT-3, *n* = 4; WT, *n* = 7; with equal sex distribution) and (**G**, **H**) triceps (UT, *n* = 8; NT-3, *n* = 9; WT, *n* = 8; with equal sex distribution) muscles from 10-month-old mice, UT cohort and treatment cohort, shown as sexes combined (grey) and separated (red: females, blue: males). Two-way ANOVA, Tukey’s multiple comparisons test. Data is represented as mean ± SEM; ^*^p < 0.05, ^**^p < 0.01, ^***^p < 0.001, ^****^p < 0.0001 showing only comparison to UT cohort.

With AAV1.NT-3 gene therapy, we observed an overall reversal of the sarcopenic effect on fiber size and alterations in fiber type composition of all muscles examined. Changes in fiber type distribution of STO, FTO, and FTG (as a percent of total) and percent loss as “sarcopenic” (compared to 10-month-old) and percent gain as “NT-3 effect” in the glycolytic (FTG) and oxidative (STO) fiber size are shown in a summary format in Figure 3. Interestingly, alterations in fiber type composition showed different patterns for anterior and posterior compartment muscles of the distal hindlimb. In the tibialis anterior muscle from males, there was a reversal of sarcopenia-related decline in STO fibers, with a notable percentage increase with treatment, along with a decrease in FTG fiber type; suggesting a switch from FTG to STO fiber type. This glycolytic to oxidative fiber type switch resulted in a significant increase in STO fiber size (from 19.76% loss to 15.03% increase). In female tibialis anterior muscle however, an opposite pattern, from oxidative to glycolytic fiber type switch was noted, which was associated with prominent FTG fiber size increase (from 13.1% loss to 18.54% increase, Figure 3A, 3B, Supplementary Table 2). Contrarily, in the gastrocnemius muscle, FTG to STO fiber type switch was notable in females along with a significant increase in STO fiber diameter (from 12. 47% loss to 20.19% increase), while neither the fiber type composition nor the fiber diameter was altered with treatment in males significantly (Figure 3C, 3D, Supplementary Tables 5, 6).

Compared to 10 months old C57BL/6 mice, the greatest size reduction in FTG and STO occurred in quadriceps muscle from 2-year-old in both sexes and was more prominent in males. With treatment, the fiber type switch from glycolytic to oxidative was associated with reversal of sarcopenic effect on oxidative fiber size, in both sexes. The size increase in FTG was meager for both sexes (Figure 3E, 3F, Supplementary Tables 8, 9). Interestingly, triceps muscle appeared more resistant to the age-related size change contrasting with other muscles. The decrease in FTG fiber diameter, compared to those from 10 months old C57BL/6 mice was milder; a size increase occurred in STO and FTO fibers in males, while the STO fiber size decrease was minimal in females (Supplementary Tables 10–12). With treatment, the fiber type switch from glycolytic to oxidative reflected as size increase for both STO and FTO fibers in both sexes, was more prominent for FTO than STO (11.3% in FTO vs. 4% in STO) in males. In addition, males showed 11.38 % size increase in FTG. A trend for STO and FTO fiber type increase was present with aging in males when untreated 2-year-old samples were compared to those from 10-month-old (Figure 3G, 3H).

**Figure 4:**
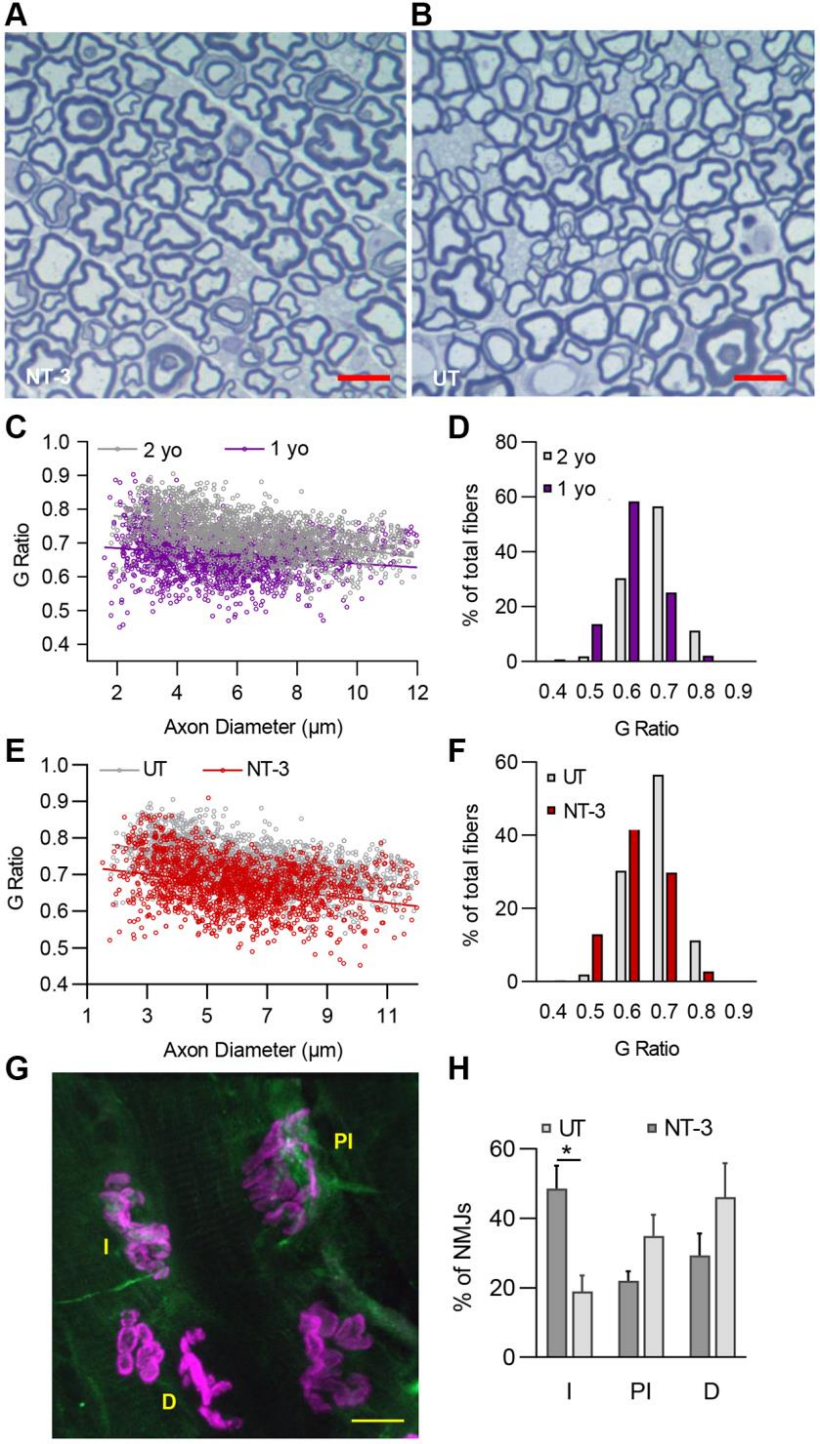
**NT-3 gene transfer improves myelin thickness of peripheral nerves and neuromuscular junction assembly in C57BL/6 mice.** Representative semithin, toluidine blue–stained cross-sections of tibial nerves from (**A**) treated and (**B**) untreated C57BL/6 mice. Scale bar = 10 μm. (**C**) Myelin thickness significantly decreased with age in 2-year-old mice compared to 1-year-old counterparts (1 yo, r^2^ = 0.0363; 2 yo, r^2^ = 0.2245; Linear regression, slopes are significantly different, *p* < 0.0001). (**D**) The shift toward thinner myelin can also be observed in percent distribution graph with aging. (**E**) A notable increase of myelin thickness was observed with treatment compared to samples from untreated (NT-3, r^2^ = 0.0989; UT, r^2^ = 0.2245; Linear regression, slopes are significantly different, *p* = 0.0037) and (**F**) percent analysis of g ratio of treated cohort displayed a distribution that peaks at 0.6 (*n* = 6 for both treated and untreated 2-year-old mice, *n* = 4 for 1-year-old control mice, with even sex distribution). (**G**) Representative image showing innervated (I), partially innervated (PI) and denervated (D) NMJs from the lumbrical muscles of the aged C57BL/6 mice. Scale bar = 10 μm. (**H**) Percent of the innervated NMJs in the treated mice was significantly higher than the untreated mice (^*^p = 0.0123). We evaluated an average of 41.2 NMJs per mouse (*n* = 4 mice for each cohort with equal sex distribution). Data is represented as mean ± SEM; Two-way ANOVA, Sidak’s multiple comparisons test; ^*^p < 0.05.

Aging process in Schwann cells, can easily be identified on the high resolution semi-thin cross sections of peripheral nerves which includes an overall thinning of myelin [17, 18] and age-related myelin alterations, such as increased myelin corrugation, infoldings and outfoldings reflecting axonal atrophy. We observed these changes in sciatic and tibial nerves distally in 2-year-old C57BL/6 mice. To assess the efficacy of NT-3 gene therapy on the aging process in peripheral nerves, we chose to evaluate G ratio (axon diameter/fiber diameter), a measure of myelin thickness in the distal tibial nerve, from aged C57BL/6 mice at 6 months post gene delivery, and compared to untreated counterparts (Figure 4A, 4B). Thinning of myelin in rodent nerves with aging has been reported previously, although mean G ratio in sciatic nerves from aged group was not found to be significantly different from younger groups, explained by the presence of concomitant axon size decrease [17–19]. In distal tibial nerves however, we found significantly increased mean G ratio in aged C57BL/6 mice, reflecting an age related thinning of myelin, when compared to data obtained from 1 year old mice (Figure 4C, 4D; 2-year-old, 0.727 ± 0.0013 vs. 1-year old, 0.665 ± 0.0015; *p* < 0.0001). The percent of fibers with a G ratio ≥ 0.7, a reflection of thin myelin in aged mice, constituted 67.79%, whereas in 1 year old mice, this was only 27.32% of total fibers. NT-3 gene therapy resulted in an attenuation of age-related myelin change, indicated by treated and untreated groups having significantly different slopes of G ratio scatterplots (Figure 4E). The mean G ratio was significantly improved toward optimal peripheral nerve G ratio of 0.6 reflecting thicker myelin at 6 months post AAV1.NT-3 gene delivery (NT-3, 0.672 ± 0015 vs. untreated, 0.727 ± 0.0013; *p* < 0.0001). The percent fibers with G ratio ≥ 0.7 representing thin myelin reduced to 32.62%, which constituted 67.79% of total fibers in the untreated cohort (Figure 4F).

We also assessed the status of NMJ in response to NT-3 gene therapy in this model as there is evidence that changes in endplate morphology and NMJ remodeling occur with aging and precede loss of fast motor units [20]. Using immunohistochemistry-based parameters [11], we analyzed a total of 330 NMJs derived from intrinsic foot muscles of NT-3 treated and untreated aged C57BL/6 mice. This analysis showed that AAV1.NT-3 gene therapy, at 6 months of treatment gave rise to a 29.7% increase of innervated NMJs (*p* = 0.0123). A decrease in the denervated and partially denervated/innervated NMJs was also noted (Figure 4G, 4H). We believe that these quantitative histopathological assessments of muscle, NMJ and nerve collectively provide strong support to the functional improvements observed in aged C57BL/6 mice with NT-3 gene therapy.

### NT-3 induced remodeling of muscle metabolism in age-related sarcopenia

**Figure 5:**
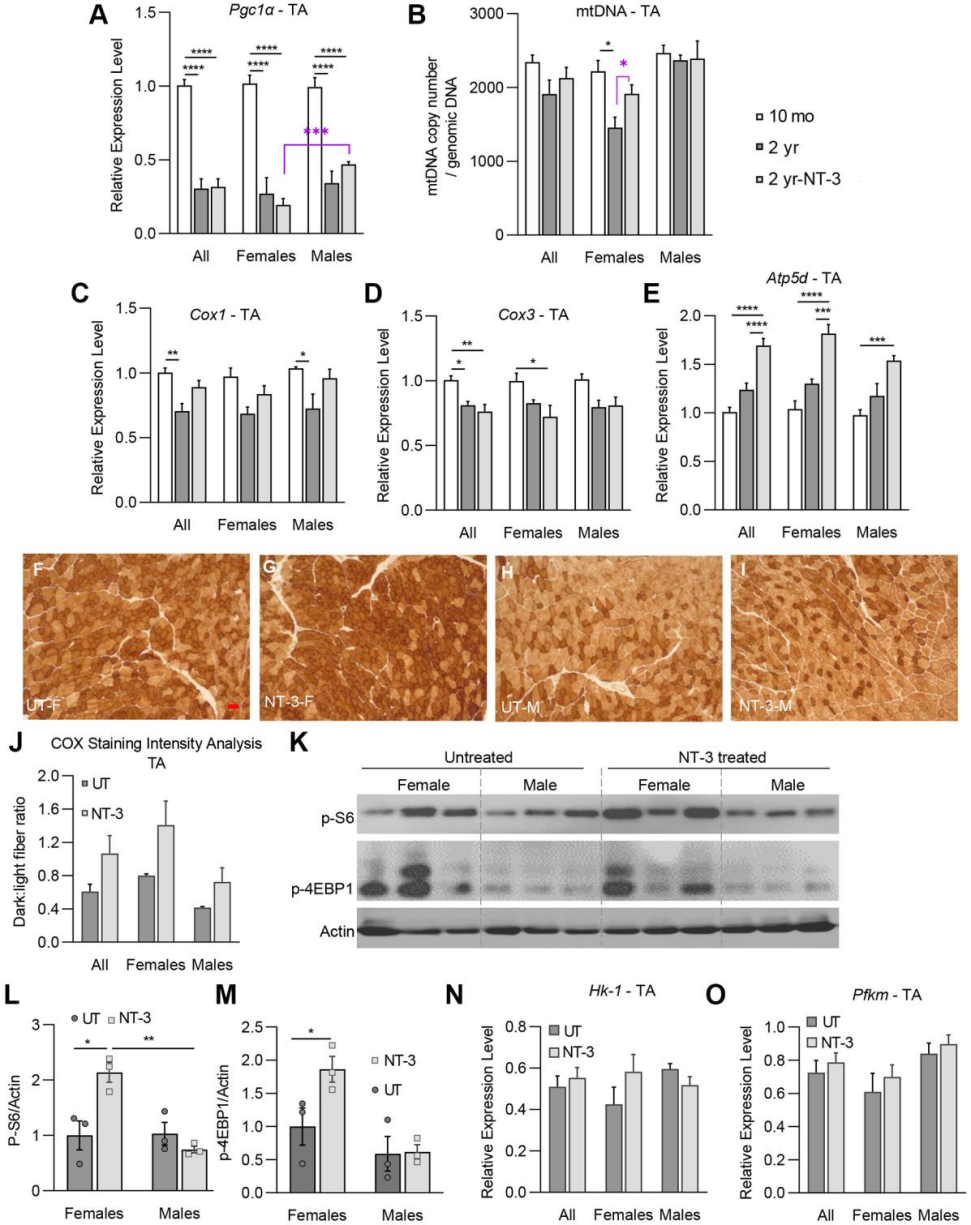
**NT-3-treatment-induced changes on markers of mitochondrial biogenesis, oxidative phosphorylation and mTORC1 pathway in tibialis anterior muscle.** Bar graphs represent (**A**) relative expression levels of *Pgc1α*, (**B**) mtDNA copy number/genomic DNA, relative expression levels of (**C**) *Cox1*, (**D**) *Cox3*, and (**E**) *Atp5d* genes of tibialis anterior muscle in treated and untreated C57BL/6 mice (*n* = 8, 10-month-old (mo) mice; *n* = 8, 2-year-old untreated mice (2 yr); *n* = 9, 2-year-old NT-3 treated (2 yr-NT-3) mice; with equal sex distribution). (**F**–**I**) Representative images of COX-stained sections of tibialis anterior muscle in the treated and untreated female and male mice. Scale bar: 25 μm, applies to all images (**J**) Bar graphs showing the intensity analysis on COX-stained sections (*n* = 6, untreated mice; *n* = 6, NT-3 treated mice; with equal sex distribution). (**K**) Western blots showing the expression level of p-S6, and p-4E-BP1 proteins. Protein levels of (**L**) p-S6, and (**M**) p-4E-BP1 normalized to Actin (*n* = 6 for both cohorts with equal sex distribution, blots were cropped for conciseness). Relative expression levels of (**N**) *Hk-1* and (**O**) *Pfkm* enzymes (*n* = 8, 2-year-old untreated mice; *n* = 9, 2-year-old NT-3 treated mice). Student *t*-test for the analysis marked with purple asterisk. Two-way ANOVA, Tukey’s multiple comparisons test. Data is represented as mean ± SEM; ^*^p < 0.05, ^**^p < 0.01, ^***^p < 0.001, ^****^p < 0.0001.

Studies have shown that mtDNA and mRNA abundance and mitochondrial ATP production, all decline with advancing age [21–23]. We first started by investigating whether the mitochondria biogenesis marker *Pgc1α* transcripts and mtDNA copy number per genomic DNA are altered in muscle at 6 months post NT-3 gene therapy in aged C57BL/6 mice. In the untreated tibialis anterior muscle, *Pgc1α* transcripts showed a significant decline in both sexes at 2 years of age, compared to 10 months old mouse samples (Figure 5A). The response to NT-3 gene therapy, however, showed sex difference; with males having significantly more *Pgc1α* transcripts than females. The mitochondria copy number in females from the untreated cohort was significantly lower than 10-month-old samples, while the treated cohort showed an increase. Interestingly, mitochondria copy number in the untreated and treated muscle from males did not differ from the 10-month-old males (Figure 5B). We then analyzed the expression levels of *Cox1* and *Cox3*, which are mtDNA encoded subunits of cytochrome c oxidase of respiratory complex IV and *Atp5d* subunit of complex V, encoded by genomic DNA. We also investigated if intensity-quantification of histochemical staining for cytochrome c oxidase (COX) enzyme activity in these muscles show any correlation with the expression levels of these subunits, and/or directly reflect the functional mitochondria content in the muscle. As expected, *Cox1* and *Cox3* transcripts significantly declined in the untreated tibialis anterior muscle at 2 years of age compared to the levels from younger C57BL/6 controls (Figure 5C, 5D). AAV1.NT-3 treatment gave rise to a trend of increased *Cox1* expression, contrasting with highly significant increases in nuclear encoded *Atp5d* transcripts in both sexes, which was more prominent in females (Figure 5C–5E). We also found COX enzyme stain-intensity quantification correlating well with increased *Atp5d* expression levels and mitochondria content, more notable in females compared to the untreated muscle (Figure 5F–5J).

Considering the significant size increase in FTG fibers, along with increased glycolytic fiber type in the female tibialis anterior muscle, we next explored the possibility of mTORC1 activation, as previously shown with NT-3 in the neurogenic muscle from trembler J mouse (Tr^J^) [9]. In western blot analysis, we found significant increases in the downstream targets of mTORC1, phosphorylated S6 kinase and 4E-BP1 proteins, indicating the activation of mTORC1 which correlate with increased radial growth of FTG fibers (Figure 5K–5M). In addition, we found a trend for increased expression of glycolytic enzymes *Pfkm* and *Hk-1* in females, suggesting increased carbohydrate metabolism in muscle. In contrast, expression of phosphorylated S6 protein and HK-1 transcripts were lower in males compared to untreated samples, in keeping with the sex-dependent pattern of changes in this muscle (Figure 5N, 5O).

**Figure 6:**
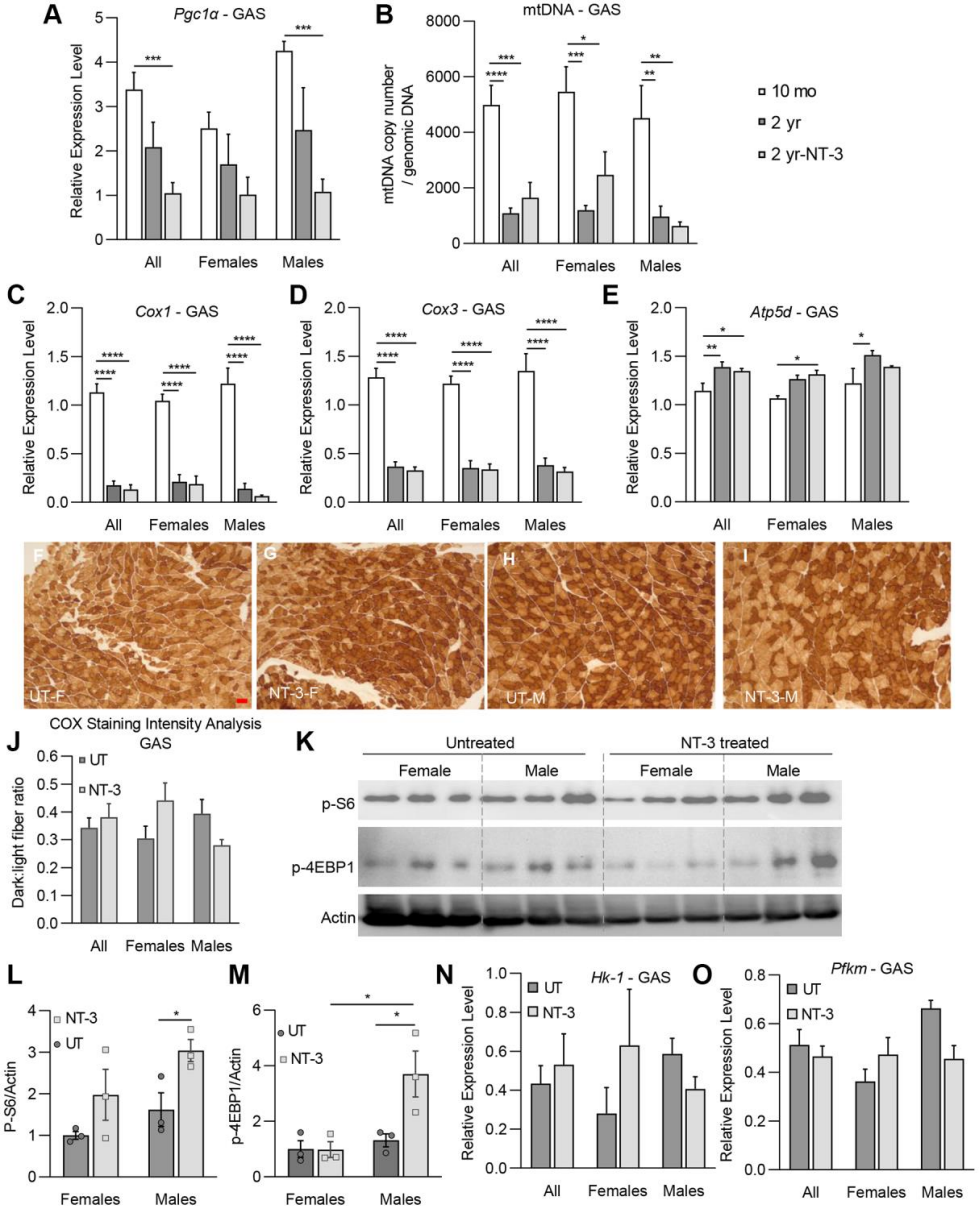
**NT-3-treatment-induced changes on markers of mitochondrial biogenesis, oxidative phosphorylation and mTORC1 pathway in gastrocnemius muscle.** Bar graphs represent (**A**) relative expression levels of Pgc1α, (**B**) mtDNA copy number/genomic DNA, relative expression levels of (**C**) *Cox1*, (**D**) *Cox3*, and (**E**) *Atp5d* genes of gastrocnemius muscle in treated and untreated aged C57BL/6 mice. (*n* = 8, 10-month-old (mo) mice; *n* = 8, 2-year-old (2 yr) untreated mice; *n* = 9, 2-year-old NT-3 treated (2 yr-NT-3) mice; with equal sex distribution). (**F**–**I**) Representative images of COX-stained sections of tibialis anterior muscle in the treated and untreated female and male mice. Scale bar: 25 μm, applies to all images (**J**) Bar graphs showing the intensity analysis on COX-stained sections (*n* = 7, untreated mice; *n* = 8, NT-3 treated mice; with equal sex distribution). (**K**) Western blots showing the expression level of p-S6, and p-4E-BP1 proteins. Protein levels of (**L**) p-S6, and (**M**) p-4E-BP1 normalized to Actin (*n* = 6 for both cohorts with equal sex distribution, blots were cropped for conciseness). Relative expression levels of (**N**) *Hk-1* and (**O**) *Pfkm* enzymes (*n* = 8, 2-year-old untreated mice; *n* = 9, 2-year-old NT-3 treated mice). Student *t*-test for part L. Two-way ANOVA, Tukey’s multiple comparisons test. Data is represented as mean ± SEM; ^*^p < 0.05, ^**^p < 0.01, ^***^p < 0.001, ^****^p < 0.0001.

We found similar changes with aging in the untreated gastrocnemius muscle at 2 years of age for both *Pgc1α* transcripts as well as the mitochondria copy number, which were significantly lower than 10-month-old counterparts (Figure 6A, 6B). At 6 months post NT-3 gene therapy, although not statistically significant, there was further decline in *Pgc1α* relative expression levels compared to untreated counterparts, without sex difference (Figure 6A). The mitochondria copy number increased in the female muscle without reaching significance, contrasting with a decline in the samples from males (Figure 6B). *Cox1* and *Cox3* transcripts from untreated muscles at 2 years of age significantly declined, while *Atp5d* expression increased compared to the levels from 10 months old C57BL/6 muscle (Figure 6C–6E). With NT-3 gene therapy, we found no significant change in the expression levels of these transcripts, although there were sex-dependent differences in staining intensity of COX enzyme in the NT-3 treated muscle, compared to Ringer’s lactate-treated controls (Figure 6F–6I). Females exhibited a stronger staining intensity with treatment, while an opposite pattern was present in males, showing stronger staining intensity in the untreated muscle (Figure 6J). The COX enzyme stain-intensity quantification reflected the changes in *Cox*1, *Cox3*, and *Atp5d* transcripts and mitochondria content between males and females, compared to untreated muscle. Contrasting with tibialis anterior, in western blot analysis, phosphorylated S6 kinase and 4E-BP1 proteins showed significant increases in males, which reflected a small radial growth only in the FTO fibers, while no size increase was noted in FTG fibers in response to treatment (Figure 6K–6M, Figure 3D, Supplementary Table 6). Moreover, the *Pfkm* and *Hk-1* transcripts were lower in males compared to untreated samples suggesting that males did not respond to NT-3 induced anabolic stimulation by increasing carbohydrate metabolism in the gastrocnemius muscle. Females, nonetheless, showed an increase trend for phosphorylated S6 kinase protein levels and the *Pfkm* and *Hk-1* transcripts without reaching significance (Figure 6L–6O).

## DISCUSSION

In this study, we provide strong evidence for the efficacy of AAV1.NT-3 gene therapy in sarcopenia and age related hypomyelination of peripheral nerves, as well as the maintenance of NMJ connectivity in 2-year-old C57BL/6 mouse, a model for natural aging. At 6 months post gene delivery, the NT-3 effects on the neuromuscular system manifested as significant histopathological improvements, reflecting upon functional performance and *in vivo* muscle physiology. Attenuation of other age related musculoskeletal and skin changes with treatment including kyphosis, dermatitis, and alopecia was also striking.

In the untreated C57BL/6 mice, detailed morphometric data from all four muscles, although confirmed the previous observations of type 2 fiber atrophy in general [24–26], revealed some differences between muscles in response to aging process. The most severe glycolytic fiber atrophy occurred in both male and female quadriceps, and the most severe oxidative fiber atrophy was present in male tibialis anterior muscles; triceps were the least affected muscle by sarcopenia. At 6 months post NT-3 gene therapy, quantitative data from all four muscles revealed an overall normalization of fiber size towards values observed in 10 months of age. In the quadriceps and triceps (proximal-extensor muscles) of the treated aged cohort, there was a switch to fatigue-resistant oxidative fibers occurred in both sexes, when compared to untreated. However, in distal hindlimb muscles (anterior compartment/flexor muscle vs. posterior compartment/extensor muscle), we found what appears to be a sex-dependent muscle remodeling. The switch from oxidative to glycolytic fiber type observed in the tibialis anterior muscle in females was associated with prominent FTG size increase. In the same muscle from males, oxidative fiber size increase was greater than glycolytic, along with an increase in oxidative fiber type. Contrary to the tibialis anterior, in the female gastrocnemius muscle, there was a switch from glycolytic to oxidative fiber type, in addition to a prominent STO size increase. We were intrigued with these observations that the most prominent muscle fiber size increase occurred in the direction of fiber type switch; to oxidative vs. to glycolytic. Interestingly however, male gastrocnemius muscle failed to show any size increase in STO or FTG, while a small FTO percentage gain was detected along in addition to a meager size increase in this fiber subtype. Overall, these observations show the presence of a muscle- and sex-dependent remodeling with aging, as well as response to NT-3 treatment.

The results from the molecular studies assessing the NT-3 effect on the oxidative state of distal hind limb muscles, accompanied by western blot analyses for mTORC1 activation are in accordance with the histologic findings. We like to emphasize here that NT-3 effect is not directed to well-differentiated or normal functioning cells, but rather is operative upon remodeled-cell metabolism that may result from a pathological process [9, 27]. In this context, it is not surprising to see NT-3, through mTORC1 activation, exerts an anabolic effect in the female tibialis anterior by increasing FTG fiber size and the percentage of glycolytic fibers, and by doing so, overcoming the age-related remodeling by normalizing the muscle towards to a younger age. Even though *Pgc1α* transcripts at this time point was found not elevated, we showed evidence that NT-3 prevented the age-related mitochondria loss by increasing mitochondria copy number towards 10-month levels. This was associated with significantly increased *Atp5d* transcripts and COX stain intensity, suggesting increased mitochondrial function. Decreased *Pgc1α* levels can be interpreted as correlative of decreased STO percentage, which was also associated with an increase in FTG percentage along with a significant size increase of FTG fibers. We believe that the total mtDNA increase in tissue might be linked to these changes in FTG subtype. In contrast, NT-3 in male tibialis anterior showed no mTORC1 activation, but preserved mitochondria content and function. This was supported by an increase in *Pgc1α* and *Atp5d* transcripts as well as COX stain intensity, which was reflected in muscle histology as fiber type switch from glycolytic, back to the oxidative levels, as in the 10-month-old samples. The findings in gastrocnemius, when contrasted with tibialis anterior muscle, appear unique, as males were less responsive to NT-3. Even though with treatment, mTORC1 was activated, the male gastrocnemius muscle failed to respond with size increase in any of the fiber types. NT-3 also did not alter mitochondria copy number, or markers of oxidative phosphorylation expression significantly. In female gastrocnemius, although did not reach to significance level (likely due to small n number), NT-3 lead to some increases in the phosphorylated S6 protein, as well as in mitochondria content, which were associated with STO greater than FTG fiber size increase, along with increased STO percentage.

Studies have shown that mtDNA and mRNA abundance and mitochondrial ATP production, all decline with advancing age [21]. PGC1α is an important transcriptional coactivator of mitochondrial biogenesis and respiration [28]. Previous studies have shown that PGC1α drives the formation of fatigue resistant STO muscle fibers [29]. Decreased *Pgc1α* expression in sarcopenic muscle from old rodents has been reported [30], as well as with aging in sedentary persons compared with physically active individuals [31, 32]. Recent transcriptomic data from skeletal muscle found reduced expression of genes related to all electron transport complexes and pyruvate dehydrogenase complexes with aging in humans [33]. Our molecular studies from untreated aged C57BL/6 mice agree with these previous reports by showing decreased *Pgc1α* expression and mtDNA content as well as reduced *Cox1* and *Cox3* transcripts compared to 10-month-old muscle. Lack of *Pgc1α* increase at 6 months post gene injection, although seemingly not fitting with the finding of increased mitochondria content in female tibialis, might be explained with the possibility of *Pgc1α* activation in an earlier time point followed by an equilibrium state. In a previous study of NT-3 gene therapy in young male trembler J (Tr^J^) mice, at 4 months post AAV.NT-3 injection we found increased *Pgc1α* expression, accompanied by increased phosphorylation levels of 4E-BP1 and S6 proteins as evidence of mTORC1 activation in the gastrocnemius muscle [9]. AAV.NT-3 treatment was capable of reversing the defective expression levels of *Pgc1α* seen in the Tr^J^ neurogenic muscle along with enhanced levels of activated 4E-BP1. In the skeletal muscle, mTORC1 can regulate mitochondrial biogenesis and metabolism through 4E-BP1/PGC1α [34]. Therefore, it is conceivable that NT-3 might also promote oxidative phosphorylation through activation of 4E-BP1 and PGC1α in the muscle. PGC1α is known to co-regulate several genes, and its expression alone is considered sufficient to increase mitochondrial mass [35]; however, it was also reported that PGC1α is dispensable for exercise induced mitochondrial biogenesis in skeletal muscle [36]. Therefore, further studies are needed to assess the link between NT-3 and *Pgc1α* expression in the sarcopenic muscle, including earlier time point experiments in this model.

Physical exercise, aerobic or anaerobic, has been well accepted to be a countermeasure for sarcopenia, but also recognized as an important practice in the prevention and treatment of not only chronic and degenerative diseases, but also age-associated multisystem diseases, including cardiovascular, neurodegenerative, chronic pulmonary diseases, cancer, diabetes, and morbid obesity. Aerobic/endurance exercise helps to maintain and improve cardiovascular fitness, respiratory function and muscle oxidative capacity, whereas strength/resistance-exercise programs primarily increase type 2 muscle fiber size, muscle strength, and function. Although the underlying sequential events that lead to these muscle adaptations are poorly understood, it has been emphasized that the mechanisms that regulate these processes involve the “quality” of skeletal muscle mitochondria [37]. Cellular antioxidant capacity and oxidative stress are postulated to be critical factors in the aging process. In one study, whole body resistance exercise training in aged men and women was shown to induce significantly higher complex IV activity and decreased oxidative stress markers, compared to before training [38]. Notably, there were no apparent changes in normal mtDNA content or mtDNA deletion products, suggesting that regular resistance exercise decreases oxidative stress, but does not affect mtDNA. It was also postulated that increases in complex IV of the electron transport chain may have an indirect antioxidant effect in older adults [39]. We think these observations are relevant to this current study regarding the similarities between exercise and NT-3-induced changes in sarcopenic muscle. COX stain intensity quantification obtained from tibialis and gastrocnemius muscles in our study appears to be a sensitive indicator of functioning mitochondria, as in the case of resistance exercise, correlating well with treadmill functional tests being more prominent in females. Activation of the mTORC1 pathway leading to radial growth in glycolytic fiber type, that we see in female tibialis muscle, is also a supportive finding. Instead, an overall increase of oxidative fibers in quadriceps and triceps of both sexes, and in female gastrocnemius muscle with NT-3, is reminiscent to the effects of aerobic/endurance exercise. Moreover, exercise induced mechanical loading has been shown to induce production of IGF-1, VEGF, and hepatocyte growth factor in osteocytes cell lines, which may play roles in regulating muscle growth [40]. In addition, muscle NT-3 levels increased by exercise training have been shown to contribute to improvement in various conditions [40–44]. Moreover, although it has not been studied in the context of sarcopenia, we predict that NT-3, via its known anti-inflammatory and immunomodulatory properties, [14, 45–47] may also have an attenuating effect on age-related inflammation; presumably a contributing factor to sarcopenia. Exercise intervention, despite its effectiveness as therapeutic regime for sarcopenia, is only available to patients or elderly who are reasonably mobile and can participate in such intervention without safety concerns. When considering the burden of sarcopenia on the lifestyle of elderly, and on the healthcare system, we believe this preclinical study is providing strong support for AAV.NT-3 gene therapy in the successful management of sarcopenia, as a serious and plausible option in the future.

## MATERIALS AND METHODS

### Animals and treatment groups

Naturally aged C57BL/6 mice were included in the study (JAX stock #000664) and all animal experiments were performed according to the guidelines approved by The Research Institute at Nationwide Children’s Hospital Animal Care and Use Committee that operates in full accordance with the Animal Welfare Act and the Health Research Extension Act (IACUC approval number = AR18-00076). 18 months old C57BL/6 (6 males and 6 females, *n* = 12) mice received 1 × 10^11^ vg dose of AAV1.tMCK.NT-3, via intramuscular (IM) injection into the gastrocnemius muscle. Age- and sex-matched C57BL/6 (6 males and 6 females, *n* = 12) mice were injected with Ringer’s lactate as controls. Mice were tested functionally with treadmill, rotarod, and *in vivo* muscle contractility assay and they were sacrificed six months post-injection by an over-dosage of xylazine/ketamine anesthesia for harvesting blood, sciatic nerves, as well as upper and lower limb muscles including lumbricals, at six months post gene injection.

### AAV1.tMCK.NT-3 vector production and potency

Construct of self-complimentary (sc) AAV serotype 1 vector with muscle specific tMCK promoter was described previously [10]. The vector was produced in our Viral Vector Core at Nationwide Children’s Hospital, Columbus (Andelyn Biosciences). Aliquots of virus were stored at −80°C until used. Serum was separated from blood samples that were collected by cardiac puncture at six months post gene injection and serum NT-3 levels were detected by ELISA as previously reported (NT-3, *n* = 9; UT, *n* = 8) [10].

### Run to exhaustion test

Run to exhaustion treadmill performance test was performed at two-, four- and six-months post injection. Mice were exercised to exhaustion via treadmill (Columbus Instruments, Exer-6M Treadmill), as described previously [15]. Mice were acclimated to the treadmill prior to data collection. Mice were run to exhaustion with increasing treadmill speed by 1 meter/min each minute, starting at an initial 7 meter per minute velocity. Lanes have a shock plate that pulses at a frequency of ~3 Hz. Mice were considered at “exhaustion” level when they were unable to re-engage the treadmill for 3 seconds after resting on the shock-plate. Run duration was recorded and used to calculate the distance ran (2 months post-injection: NT-3, *n* = 12; UT, *n* = 11; 4 months post-injection, NT-3, *n* = 12; UT, *n* = 11; 6 months post-injection: NT-3, *n* = 11; UT, *n* = 8).

### *In vivo* muscle contractility assay

*In vivo* muscle contractility assay was performed at the endpoint, as described previously [15]. Hind paw of the anesthetized mouse was placed on footplate, which was attached to a dual-mode lever, and the tibia was aligned perpendicular to the lever. Subcutaneous EMG electrodes were used to stimulate the tibial nerve. Gastrocnemius muscle torque around the ankle joint was measured by muscle physiology apparatus (Aurora Scientific, ON, Canada) using isometric contraction (maximum twitch response) and fatigue (maximum tetanic response) protocols (Max twitch: NT-3, *n* = 11; UT, *n* = 8; Max tetanic: NT-3, *n* = 10; UT, *n* = 9).

### Rotarod

Rotarod test was performed at six months post-injection to assess motor function and balance of the mice. Mice were acclimated to rotarod apparatus (Columbus Instruments, Ohio, USA) prior to data collection. Rotarod protocol included a 5-rpm run with a constant acceleration of 0.2 rpm/s. The averages of the best two out of three runs were calculated (NT-3, *n* = 10; UT, *n* = 8).

### Musculoskeletal and skin changes

Age-related musculoskeletal and skin changes including kyphosis, dermatitis, and alopecia were documented semi-quantitatively. Six mice, from both treated and untreated cohorts, were photographed at the end point and the severity of kyphosis, dermatitis, and alopecia were scored. Severe changes were scored as 1, mild-moderate changes as 0.5, and no changes as 0. For kyphosis, arbitrary lines were drawn tangential to the scoliosis curvature to delineate the scoliosis angle (Supplementary Figure 3A). Angle range 90°–110° is considered as severe, 11°–150° as mild-moderate and >150° as none.

### Histological analysis

#### Muscle histology

Tibialis anterior, gastrocnemius, quadriceps, and triceps muscles from treated and untreated mice were collected and 12 μm thick cross cryostat-sections were cut. Succinic dehydrogenase (SDH) enzyme histochemistry was performed as previously described to assess metabolic fiber type distribution and myofiber size changes in the aging muscle [15]. Deep, intermediate, and superficial zones of the muscles were analyzed to represent fibers at various oxidative states equally. One representative area from each zone were photographed at 20× magnification using an Olympus BX41 microscope and SPOT Insight 12 Mp sCMOS camera. Sample selection was based on the suitability of the tissue sections, including staining quality, contrast, and lack of artifacts and not based on outcomes of behavioral or physiological analyses. Shortest distance across the muscle fiber was measured as fiber diameter (Zeiss Axiovision LE4 software V4.9.1.0) and mean fiber diameter (mean ± SEM) was calculated for each fiber type (STO, FTO, FTG) as well as for combination of all fiber types. Fiber type percent distribution of total fibers was determined for each mouse from each treatment group. Data were obtained from a total of 2747 (*n* = 9), 2959 (*n* = 9), 1722 (*n* = 5), and 1357 (*n* = 4) fibers of the treated cohort, and 2815 (*n* = 8), 2557 (*n* = 8), 2040 (*n* = 6), and 2164 (*n* = 6) fibers of the untreated cohort for tibialis anterior, triceps, gastrocnemius and quadriceps muscles respectively.

#### G ratio of the myelinated fibers

G ratio was calculated to assess the myelin thickness of fibers in tibialis nerve, as described previously [48]. Semithin, toluidine blue-stained cross sections of tibial nerve were prepared for each mouse (*n* = 6 for both treated and untreated 2-year-old mice, *n* = 4 for 1-year-old control mice, with even sex distribution) and three randomly selected nonoverlapping areas were photographed randomly at 100× magnification using an Olympus BX41 microscope and SPOT Insight 12Mp sCMOS camera. Myelin interior and exteriors were outlined in Axiovision (AxioVs40 × 64 V 4.9.1.0) to determine the area, which was used to calculate diameters to determine g ratio. A total of 1833 fibers for treated, 1928 for untreated and 1658 for 1-year-old control mice were measured to generate scattergrams and the percent g ratio distribution histograms. Slopes of treated vs. untreated and 1-year-old vs. 2-year-old mice were compared using GraphPad Prism (9.0.0).

### Immunohistochemical analysis of neuromuscular junctions (NMJ)

Lumbrical muscles collected from treated and untreated mice (*n* = 4 for both cohorts with equal sex distribution) were processed as described previously [11]. Muscles were fixed and stained with primary antibodies (Acetylcholine receptor (AChR) antibody, α Bungarotoxin, T1175, 1:500; Anti Neurofilament 200 antibody, N4142, 1:500; SV2 antibody, AB_2315387, 1:50) [49, 50], followed by incubation with secondary antibodies (Alexa Fluor 488 conjugated anti-rabbit and anti-mouse IgG, 1:500). Samples were imaged at 60× magnification using a Zeiss LSM 800 confocal microscope. NMJs were considered to be innervated when nerve completely overlapped the AChRs, as partially innervated when some parts of the AChRs were not overlapping with nerve, and as denervated when there was not any nerve co localizing with AChRs [11, 51]. An average of 41.3 NMJs per mouse were evaluated from NT-3-treated and UT-mice (*n* = 4 mice per group with equal sex distribution).

### Expression analysis

#### Protein extraction and western blot analysis

Twenty micrometer thick sections from frozen TA and GAS muscle blocks (20 section per block, *n* = 3 per group) were put into 2 ml centrifuge tubes and homogenized in homogenization buffer [125 mM Tris-HCL pH6.8, 4% SDS, 4 M Urea solution with 1X Halt protease inhibitor (ThermoFisher) and 1× phosphatase inhibitor (Sigma)] using a disposable pestle. The lysate was then incubated on a rotary spin cycle at 4°C for 2 hours, followed by centrifugation at 10,000 g for 10 min at 4°C. The supernatant was then transferred to a new tube.

Protein samples were run in Novex 10–20% Tricine mini protein gel (ThermoFisher) and transferred to PDVF membranes (GE Healthcare). Membranes were blocked for 1 h at room temperature with 5% milk in TBS-T (TBS buffer with 0.05% Tween-20). Membranes were then incubated with primary antibodies in TBS-T buffer overnight at 4°C. After 5 min of three times wash with TBS-T, membranes were incubated with secondary antibodies in 5% milk in TBS-T for 1 h. Membranes were washed again with TBS-T for 3 times and TBS for 2 times with 5 min each wash. ECL WesternSure premium chemiluminescent substrate (LI-COR) was used for band detection followed by exposure using Chemidoc Imaging system (Bio-Rad) and band intensities were quantified using ImageJ (NIH). Primary antibodies: anti-phospho S6 protein Ser235/236 (#4858), and anti-phospho 4EBP1 thr37/46 (#2855) were from Cell Signaling Technology and anti-actin antibody (sc-47778) was from Santa Cruz; secondary antibody: HRP-linked anti-rabbit/mouse IgG (#7074/7076) was purchased from Cell Signaling Technology.

#### RNA isolation and mRNA expression

Total RNA was extracted from frozen muscle blocks (20 micrometer thick sections, 20 section per block; NT-3, *n* = 9; UT, *n* = 8; WT, *n* = 8) using Mini RNeasy Plus Universal Kit (Qiagen). cDNAs were synthesized using ProtoScript II First Strand cDNA Synthesis Kit (BioLabs). Primer sets (synthesized by IDT) for *Pgc-1α*, *Cox1*, *Cox3* and *Atp5d* were obtained from previous publications [52–54] and new primers were designed for *Pfkm (F-GAAGATACCAACTCGGACCAC, R-ATGACCCATGAAGAGCATCA)* and *Hk-1* genes *(F-CGGAATGGGGAGCCTTTGG, R-GCCTTCCTTATCCGTTTCAATGG)*. All qPCR were performed using PowerUp SYBR Green Master Mix (ThermoFisher) according to the manufacturer’s instructions. qPCR assays were performed using QuantStudio 6 Flex (Applied Biosystem). Expression data were normalized to mouse *Gapdh* mRNA level and data were analyzed by ΔΔCt method.

### COX staining density analysis

Histochemical enzyme activity of cytochrome c oxidase (COX) was assessed by quantifying the COX stain intensity in tibialis anterior and gastrocnemius muscles from treated and untreated mice. 12 μm thick-fresh frozen sections were stained using COX enzyme histochemistry protocol established in our clinical neuromuscular pathology laboratory. Muscle sections representing deep, intermediate, and superficial zones from gastrocnemius (*n* = 8 treated, *n* = 7 untreated) and tibialis anterior (*n* = 6 treated, *n* = 6 untreated) muscles were photographed at 10× magnification, using an Olympus BX41 microscope and SPOT camera, with even distribution of sex. Image data were collated and processed utilizing the Python programming language. [55] Images were calibrated using representative selections from each image to establish ranges of intensity for each fiber-type; dark (oxidative/higher mitochondria content/type 1) and light (glycolytic/lower mitochondria content/type 2) fibers, including COX deficient pale fibers. Ratio of dark to light fibers was calculated and presented as mean ± SEM.

### Statistics

Adequate sample size was determined according to our previous studies that performed analogous experiments [11, 15]. All statistical analyses were performed in GraphPad Prism 9.0 software. Two tail Student *t*-test, one-way ANOVA with Tukey’s multiple comparison test, two-way ANOVA with Sidak’s multiple comparison test or linear regression analysis were performed when applicable, and significance level was set at *P* ≤ 0.05. The tests that meet the best assumptions of the data were chosen. Results were given as mean ± SEM in all experiments and the number of animals was mentioned in figure legends along with the name of the statistical analysis performed. Other than the functional tests and quantitative muscle analysis, no blinding was used.

## Supplementary Materials

Supplementary Figures

Supplementary Tables
