# Supplementary material for: AAV1.NT-3 gene therapy prevents age-related sarcopenia
Source: Aging (Albany NY). 2023 Mar 9;15(5):1306–29. doi: 10.18632/aging.204577 (PMC10042697; doi:10.18632/aging.204577)
Supplement: Supplementary Figures [file aging-15-204577-s001.pdf]

SUPPLEMENTARY FIGURES

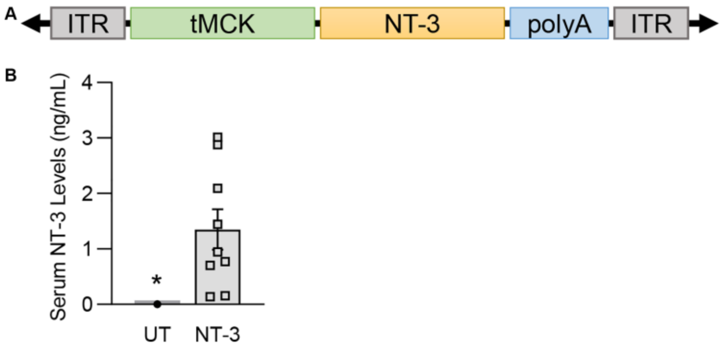

Supplementary Figure 1. scAAV1.tMCK.NT-3 vector and serum NT-3 levels. (**A**) The diagram shows the NT-3 expression cassette composed of a tMCK enhancer/promoter region (714 bp), the full-length NT-3 cDNA (774 bp) and the SV40 polyA tail (211 bp). Diagram is modified from Ozes et al., 2021 [16]. (**B**) 18 months old C57BL/6 mice were injected with 1 × 10^{11} vg of scAAV1.tMCK.NT-3 vector, or with Ringer's lactate as control in the right gastrocnemius muscle. At endpoint, serum samples were obtained from treated (NT-3) and untreated (UT) mice via cardiac puncture, and NT-3 levels were determined by ELISA. Error bars are ± SEM; *n* = 9 for treated cohort. Asterisk depicts UT mice (*n* = 8) with NT-3 serum levels below detection range.

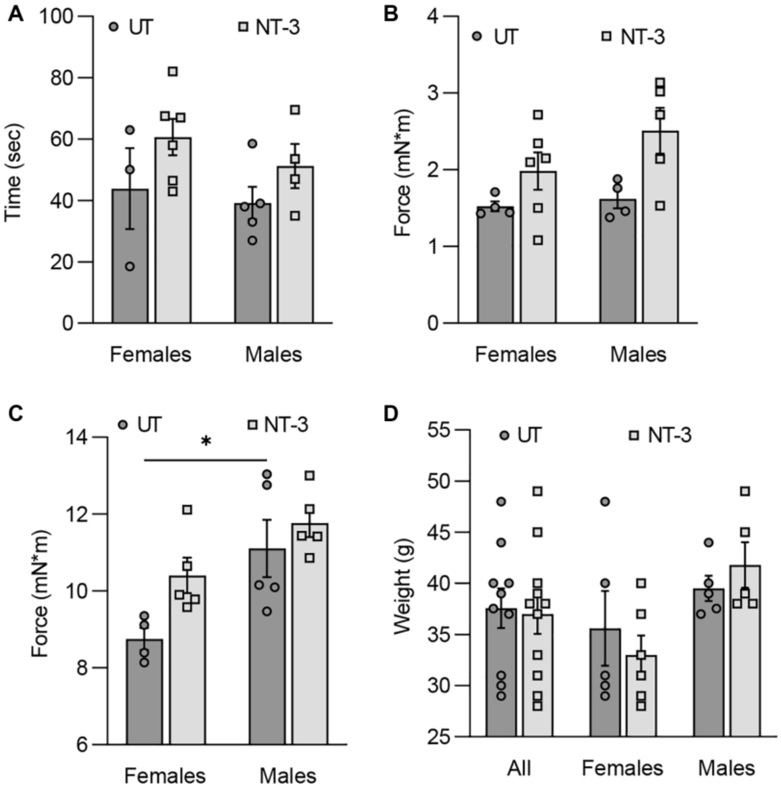

Supplementary Figure 2. Rotarod and *in vivo* muscle physiology data from 2-year-old male and female C57BL/6 mice. (**A**) The bar graphs show sex-separated rotarod data at end point (Females: NT-3, 60.67 ± 7.80 sec, *n* = 6; UT, 43.83 ± 15.19 sec, *n* = 3; Males: NT-3, 51.25 ± 8.90 sec, *n* = 4; UT, 39.10 ± 7.07 sec, *n* = 5). Sex-separated *in vivo* muscle contractility data for (**B**) maximum twitch (Females: NT-3, 1.984 ± 0.24 mN*m, *n* = 6; UT, 1.523 ± 0.06 mN*m, *n* = 4; Males: NT-3, 2.509 ± 0.30 mN*m, *n* = 5; UT, 1.619 ± 0.12 mN*m, *n* = 4) and (**C**) maximum tetanic responses (Females: NT-3, 10.40 ± 1.04 mN*m, *n* = 5; UT, 8.75 ± 0.58 mN*m, *n* = 4; Males: NT-3, 11.77 ± 0.82 mN*m, *n* = 5; UT, 11.11 ± 1.66 mN*m, *n* = 5). (**D**) The treatment did not alter the endpoint weight of the treated cohort (All: NT-3, 37.0 ± 1.95 g, *n* = 11; UT, 37.6 ± 1.94, *n* = 10 g; Females: NT-3, 33.0 ± 1.91 g, *n* = 6; UT, 35.6 ± 3.67 g, *n* = 5; Males: NT-3, 41.8 ± 2.22 g, *n* = 5; UT, 39.5 ± 1.24 g, *n* = 5). Data is represented as mean ± SEM; Two-way ANOVA, Tukey's multiple comparisons test for (**A–C**) and Sidak's multiple comparisons test for (**D**); $^{*}p < 0.05$.

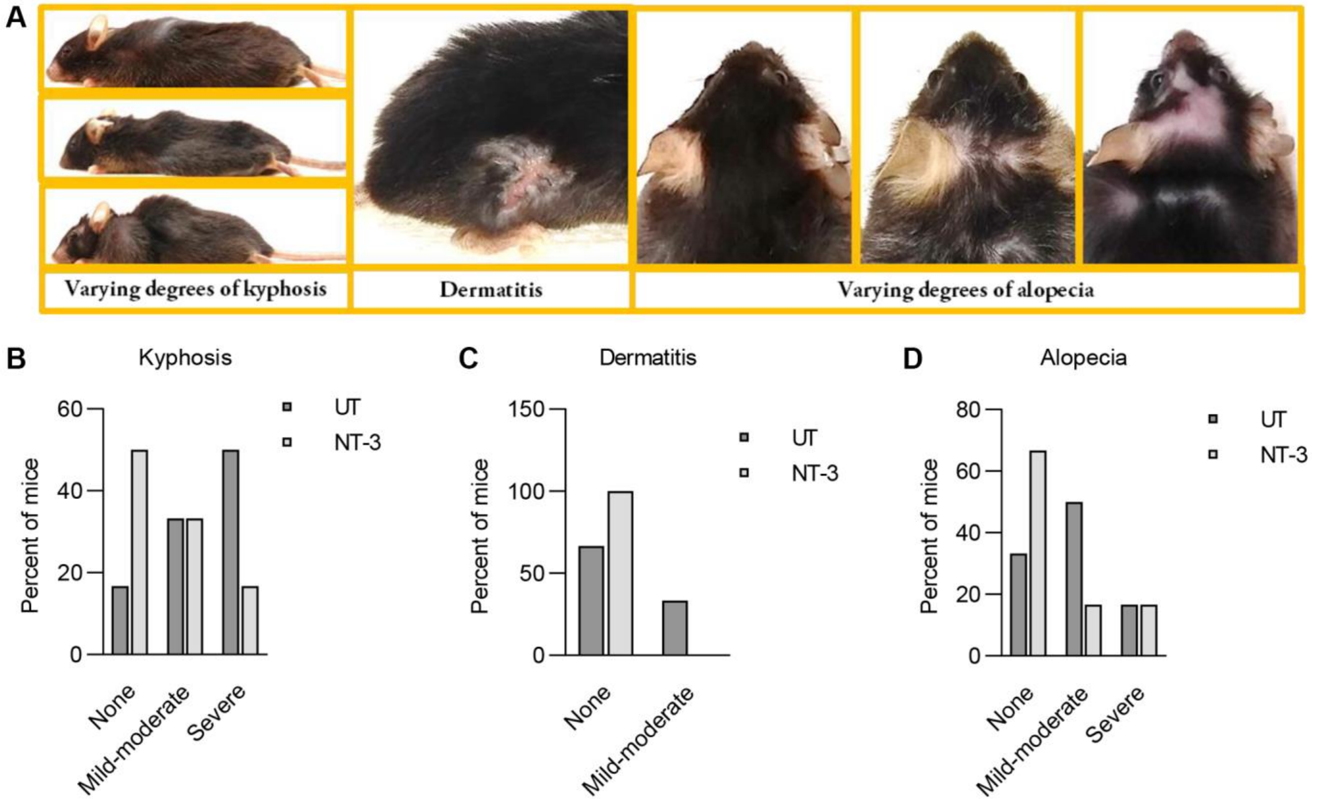

Supplementary Figure 3. NT-3 improves age-related musculoskeletal and skin changes. (**A**) Representative photographs of C57BL/6 mice with various degrees of kyphosis, dermatitis, and alopecia. (**B**–**D**) Bar graphs display the percent distribution of the musculoskeletal and skin changes calculated based on the severity scores (0: none, 0.5: mild-moderate, 1: severe). (**B**) Kyphosis scoring suggested that 50% of the untreated mice had severe kyphosis while this ratio decreased to 16.7% in the treated group. (**C**) Dermatitis was not observed in the NT-3 treated cohort however 33% of untreated mice had mild-moderate dermatitis. (**D**) Percent of the moderate to severe alopecia was 67% in the untreated mice whereas this ratio reduced to 33% with treatment (n = 6 for each cohort).

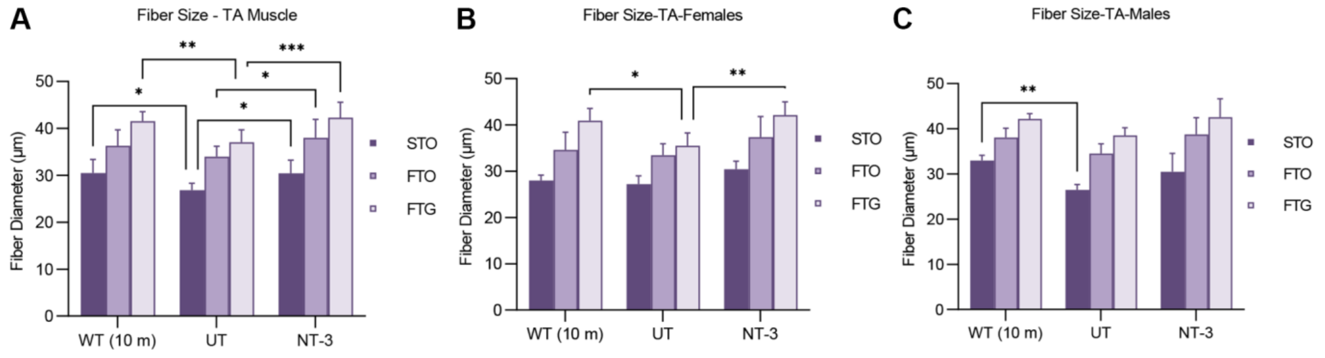

Supplementary Figure 4. Fiber size analysis of the tibialis anterior muscle. Fiber size measurements of the tibialis anterior muscle from 10-month-old (mo), 2-year-old untreated and 2-year-old NT-3-treated C57BL/6 mice shown as (**A**) combined, (**B**) females only, and (**C**) males only. Each bar represents mean ± SEM. $^{*}p < 0.05$, $^{**}p < 0.01$, $^{***}p < 0.001$, 2-way ANOVA, Tukey's multiple comparison test.

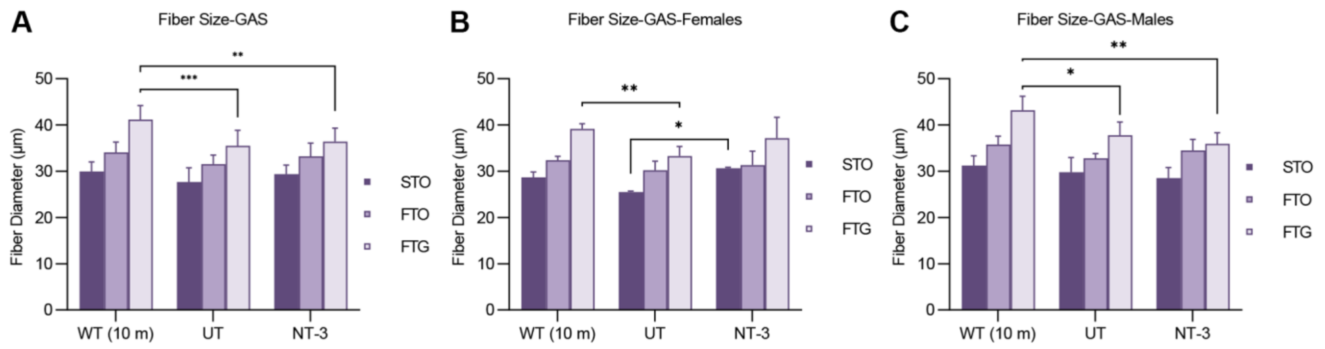

Supplementary Figure 5. Fiber size analysis of the gastrocnemius muscle. Fiber size measurements of the gastrocnemius muscle from 10-month-old (mo), 2-year-old untreated and 2-year-old NT-3-treated C57BL/6 mice shown as (**A**) combined, (**B**) females only, and (**C**) males only. Each bar represents mean ± SEM. $^{*}p < 0.05$, $^{**}p < 0.01$, $^{***}p < 0.001$, 2-way ANOVA, Tukey's multiple comparison test.

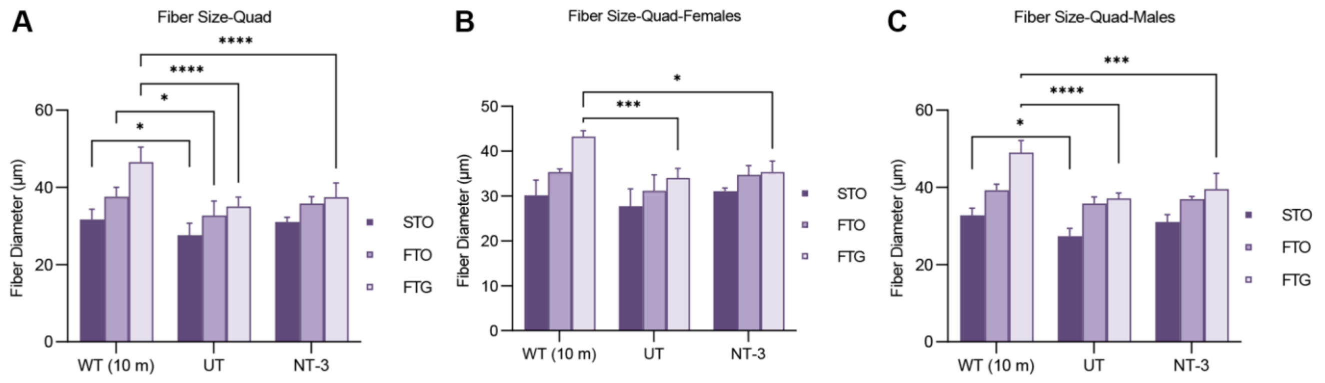

Supplementary Figure 6. Fiber size analysis of the quadriceps muscle. Fiber size measurements of the quadriceps muscle from 10-month-old (mo), 2-year-old untreated and 2-year-old NT-3-treated C57BL/6 mice shown as (**A**) combined, (**B**) females only, and (**C**) males only. Each bar represents mean ± SEM. $^{*}p < 0.05$, $^{**}p < 0.01$, $^{***}p < 0.001$, 2-way ANOVA, Tukey's multiple comparison test.

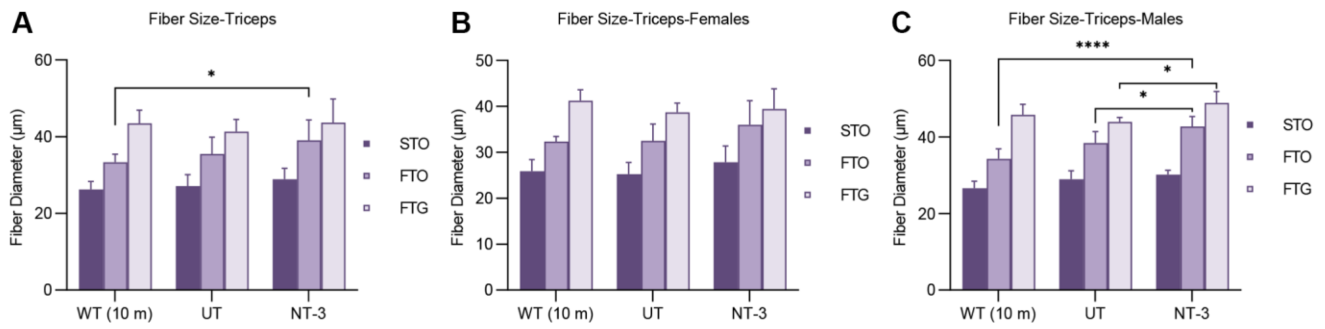

Supplementary Figure 7. Fiber size analysis of the triceps muscle. Fiber size measurements of the triceps muscle from 10-month-old (mo), 2-year-old untreated and 2-year-old NT-3-treated C57BL/6 mice shown as (**A**) combined, (**B**) females only, and (**C**) males only. Each bar represents mean ± SEM. $^{*}p < 0.05$, $^{**}p < 0.01$, $^{***}p < 0.001$, $^{****}p < 0.0001$, 2-way ANOVA, Tukey's multiple comparison test.
