# Supplementary material for: AAV1.NT-3 gene therapy prevents age-related sarcopenia
Source: Aging (Albany NY). 2023 Mar 9;15(5):1306–29. doi: 10.18632/aging.204577 (PMC10042697; doi:10.18632/aging.204577)
Supplement: Supplementary Tables [file aging-15-204577-s002.pdf]

SUPPLEMENTARY TABLES

Supplementary Table 1. Fiber size analysis on tibialis anterior muscle of NT-3 treated and untreated C57BL/6 mice.

	WT (10 mo, *n* = 8)	Untreated (*n* = 8)	NT-3 Treated (*n* = 9)
	Diameter (µm)	Diameter (µm)	Diameter (µm)
STO	30.49 ± 1.0	26.85 ± 0.5$^{\#}$	30.43 ± 0.9*
FTO	36.33 ± 1.1	33.97 ± 0.8	37.98 ± 1.3*
FTG	41.54 ± 2.0	37.08 ± 1.0$^{\#\#}$	42.31 ± 1.1***
All Fibers	37.23 ± 0.9	33.27 ± 0.8$^{\#}$	37.46 ± 0.9**

Data represented as mean ± SEM. $^{*}p < 0.05$, $^{**}p < 0.01$, $^{***}p < 0.001$, $^{****}p < 0.0001$ (compared to UT), $^{\#}p < 0.05$, $^{\#\#}p < 0.01$ (compared to WT-10 mo), 2-way ANOVA, Tukey's multiple comparison test.

Supplementary Table 2. Fiber size analysis on tibialis anterior muscle of NT-3 treated and untreated C57BL/6 female mice.

	WT (10 mo, *n* = 4)	Untreated (*n* = 4)	NT-3 Treated (*n* = 5)
	Diameter (µm)	Diameter (µm)	Diameter (µm)
STO	27.98 ± 0.5	27.22 ± 0.9	30.41 ± 0.8
FTO	34.61 ± 1.7	33.44 ± 1.3	37.79 ± 2.0
FTG	40.89 ± 1.2	35.54 ± 1.4$^{\#}$	42.13 ± 1.3**
All Fibers	35.26 ± 0.9	32.20 ± 1.0	36.95 ± 1.1*

Data represented as mean ± SEM. $^{*}p < 0.05$, $^{**}p < 0.01$, $^{***}p < 0.001$, $^{****}p < 0.0001$ (compared to UT), $^{\#}p < 0.05$, $^{\#\#}p < 0.01$ (compared to WT-10 mo), 2-way ANOVA, Tukey's multiple comparison test.

Supplementary Table 3. Fiber size analysis on tibialis anterior muscle of NT-3 treated and untreated C57BL/6 male mice.

	WT (10 mo, *n* = 4)	Untreated (*n* = 4)	NT-3 Treated (*n* = 4)
	Diameter (µm)	Diameter (µm)	Diameter (µm)
STO	33.00 ± 0.5	26.48 ± 0.6$^{\#\#}$	30.46 ± 2.0
FTO	38.04 ± 0.9	34.50 ± 1.1	38.72 ± 1.9
FTG	42.19 ± 0.5	38.56 ± 0.8	42.54 ± 2.1
All Fibers	39.20 ± 0.4	34.33 ± 0.9$^{\#}$	38.09 ± 1.7

Data represented as mean ± SEM. $^{*}p < 0.05$, $^{**}p < 0.01$, $^{***}p < 0.001$, $^{****}p < 0.0001$ (compared to UT), $^{\#}p < 0.05$, $^{\#\#}p < 0.01$ (compared to WT-10 mo), 2-way ANOVA, Tukey's multiple comparison test.

Supplementary Table 4. Fiber size analysis on gastrocnemius muscle of NT-3 treated and untreated C57BL/6 mice.

	WT (10 mo, *n* = 8)	Untreated (*n* = 6)	NT-3 Treated (*n* = 5)
	Diameter (µm)	Diameter (µm)	Diameter (µm)
STO	29.96 ± 0.7	27.65 ± 1.3	29.39 ± 0.9
FTO	34.07 ± 0.7	31.53 ± 0.8	33.23 ± 1.3
FTG	41.19 ± 1.0	35.54 ± 1.3$^{\#\#\#}$	36.43 ± 1.3$^{\#\#}$
All Fibers	36.51 ± 0.8	32.52 ± 1.2$^{\#}$	33.66 ± 0.8

Data represented as mean ± SEM. $^{*}p < 0.05$, $^{**}p < 0.01$, $^{***}p < 0.001$, $^{****}p < 0.0001$ (compared to UT), $^{\#}p < 0.05$, $^{\#\#}p < 0.01$ (compared to WT-10 mo), 2-way ANOVA, Tukey's multiple comparison test.

Supplementary Table 5. Fiber size analysis on gastrocnemius muscle of NT-3 treated and untreated C57BL/6 female mice.

	WT (10 mo, n = 4)	Untreated (n = 3)	NT-3 Treated (n = 2)
	Diameter (μm)	Diameter (μm)	Diameter (μm)
STO	28.69 ± 0.5	25.51 ± 0.1	30.66 ± 0.1*
FTO	32.67 ± 0.3	30.24 ± 1.1	31.33 ± 2.2
FTG	39.17 ± 0.5	33.30 ± 1.2$^{\#\#}$	37.18 ± 3.2
All Fibers	34.97 ± 0.5	30.35 ± 0.8$^{\#\#}$	33.59 ± 1.7

Data represented as mean ± SEM. $^{*}p < 0.05$, $^{**}p < 0.01$, $^{***}p < 0.001$, $^{****}p < 0.0001$ (compared to UT), $^{\#}p < 0.05$, $^{\#\#}p < 0.01$ (compared to WT-10 mo), 2-way ANOVA, Tukey's multiple comparison test.

Supplementary Table 6. Fiber size analysis on gastrocnemius muscle of NT-3 treated and untreated C57BL/6 male mice.

	WT (10 mo, n = 4)	Untreated (n = 3)	NT-3 Treated (n = 3)
	Diameter (μm)	Diameter (μm)	Diameter (μm)
STO	31.24 ± 0.9	29.79 ± 1.8	28.55 ± 1.3
FTO	36.32 ± 1.0	32.82 ± 0.6	34.50 ± 1.4
FTG	43.20 ± 1.3	37.78 ± 1.7$^{\#}$	35.94 ± 1.4$^{\#\#}$
All Fibers	38.04 ± 0.9	34.70 ± 1.2	33.71 ± 1.2

Data represented as mean ± SEM. $^{*}p < 0.05$, $^{**}p < 0.01$, $^{***}p < 0.001$, $^{****}p < 0.0001$ (compared to UT), $^{\#}p < 0.05$, $^{\#\#}p < 0.01$ (compared to WT-10 mo), 2-way ANOVA, Tukey's multiple comparison test.

Supplementary Table 7. Fiber size analysis on quadriceps muscle of NT-3 treated and untreated C57BL/6 mice.

	WT (10 mo, n = 7)	Untreated (n = 6)	NT-3 Treated (n = 4)
	Diameter (μm)	Diameter (μm)	Diameter (μm)
STO	31.66 ± 1.0	27.60 ± 1.3$^{\#}$	31.06 ± 0.6
FTO	37.59 ± 0.8	32.72 ± 1.5$^{\#}$	35.83 ± 0.9
FTG	46.56 ± 1.4	35.06 ± 1.0$^{\#\#\#\#}$	37.47 ± 1.8$^{\#\#\#\#}$
All Fibers	42.02 ± 1.3	33.35 ± 1.0$^{\#\#\#\#}$	35.11 ± 1.2$^{\#\#}$

Data represented as mean ± SEM. $^{*}p < 0.05$, $^{**}p < 0.01$, $^{***}p < 0.001$, $^{****}p < 0.0001$ (compared to UT), $^{\#}p < 0.05$, $^{\#\#}p < 0.01$ (compared to WT-10 mo), 2-way ANOVA, Tukey's multiple comparison test.

Supplementary Table 8. Fiber size analysis on quadriceps muscle of NT-3 treated and untreated C57BL/6 female mice.

	WT (10 mo, n = 3)	Untreated (n = 4)	NT-3 Treated (n = 2)
	Diameter (μm)	Diameter (μm)	Diameter (μm)
STO	30.19 ± 1.4	27.72 ± 1.9	31.06 ± 0.5
FTO	35.36 ± 0.3	31.19 ± 1.8	34.73 ± 1.5
FTG	43.23 ± 0.6	34.01 ± 1.1	35.37 ± 1.7
All Fibers	38.87 ± 1.0	32.55 ± 1.3$^{\#}$	34.09 ± 0.9

Data represented as mean ± SEM. $^{*}p < 0.05$, $^{**}p < 0.01$, $^{***}p < 0.001$, $^{****}p < 0.0001$ (compared to UT), $^{\#}p < 0.05$, $^{\#\#}p < 0.01$ (compared to WT-10 mo), 2-way ANOVA, Tukey's multiple comparison test.

Supplementary Table 9. Fiber size analysis on quadriceps muscle of NT-3 treated and untreated C57BL/6 male mice.

	WT (10 mo, *n* = 4)	Untreated (*n* = 2)	NT-3 Treated (*n* = 2)
	Diameter (μm)	Diameter (μm)	Diameter (μm)
**STO**	32.77 ± 0.8	27.35 ± 1.4	31.07 ± 1.3
**FTO**	40.61 ± 0.2	35.80 ± 1.2	36.94 ± 0.5
**FTG**	49.06 ± 1.3	37.16 ± 1.0	39.58 ± 2.9
**All Fibers**	44.38 ± 0.9	34.95 ± 0.4$^{###}$	36.14 ± 2.3$^{##}$

Data represented as mean ± SEM. $^{*}p < 0.05$, $^{**}p < 0.01$, $^{***}p < 0.001$, $^{****}p < 0.0001$ (compared to UT), $^{\#}p < 0.05$, $^{\#\#}p < 0.01$ (compared to WT-10 mo), 2-way ANOVA, Tukey's multiple comparison test.

Supplementary Table 10. Fiber size analysis on triceps muscle of NT-3 treated and untreated C57BL/6 mice.

	WT (10 mo, *n* = 8)	Untreated (*n* = 8)	NT-3 Treated (*n* = 9)
	Diameter (μm)	Diameter (μm)	Diameter (μm)
**STO**	26.22 ± 0.7	27.10 ± 1.1	28.87 ± 1.0
**FTO**	33.32 ± 0.7	35.46 ± 1.6	39.01 ± 1.8$^{\#}$
**FTG**	43.50 ± 1.1	41.31 ± 1.1	43.65 ± 2.1
**All Fibers**	37.70 ± 0.8	35.92 ± 1.2	37.18 ± 1.6

Data represented as mean ± SEM. $^{*}p < 0.05$, $^{**}p < 0.01$, $^{***}p < 0.001$, $^{****}p < 0.0001$ (compared to UT), $^{\#}p < 0.05$, $^{\#\#}p < 0.01$ (compared to WT-10 mo), 2-way ANOVA, Tukey's multiple comparison test.

Supplementary Table 11. Fiber size analysis on triceps muscle of NT-3 treated and untreated C57BL/6 female mice.

	WT (10 mo, *n* = 4)	Untreated (*n* = 4)	NT-3 Treated (*n* = 5)
	Diameter (μm)	Diameter (μm)	Diameter (μm)
**STO**	25.88 ± 1.1	25.21 ± 1.3	27.85 ± 1.6
**FTO**	35.35 ± 0.5	32.51 ± 1.8	36.02 ± 2.3
**FTG**	41.22 ± 1.0	38.69 ± 1.0	39.43 ± 2.0
**All Fibers**	35.96 ± 1.0	33.17 ± 1.1	33.90 ± 1.3

Data represented as mean ± SEM. $^{*}p < 0.05$, $^{**}p < 0.01$, $^{***}p < 0.001$, $^{****}p < 0.0001$ (compared to UT), $^{\#}p < 0.05$, $^{\#\#}p < 0.01$ (compared to WT-10 mo), 2-way ANOVA, Tukey's multiple comparison test.

Supplementary Table 12. Fiber size analysis on triceps muscle of NT-3 treated and untreated C57BL/6 male mice.

	WT (10 mo, *n* = 4)	Untreated (*n* = 4)	NT-3 Treated (*n* = 4)
	Diameter (μm)	Diameter (μm)	Diameter (μm)
**STO**	26.57 ± 0.8	28.98 ± 1.1	30.14 ± 0.6
**FTO**	34.29 ± 1.1	38.41 ± 1.5	42.75 ± 1.3$^{*\#\#\#\#}$
**FTG**	45.77 ± 1.2	43.93 ± 0.6	48.93 ± 1.5*
**All Fibers**	39.45 ± 0.6	38.66 ± 0.8	41.26 ± 1.2

Data represented as mean ± SEM. $^{*}p < 0.05$, $^{**}p < 0.01$, $^{***}p < 0.001$, $^{****}p < 0.0001$ (compared to UT), $^{\#}p < 0.05$, $^{\#\#}p < 0.01$ (compared to WT-10 mo), 2-way ANOVA, Tukey's multiple comparison test.
